# Stochastic resonance stimulation improves balance in children with cerebral palsy: a case control study

**DOI:** 10.1186/s12984-018-0467-7

**Published:** 2018-12-10

**Authors:** Anastasia Zarkou, Samuel C. K. Lee, Laura A. Prosser, Sungjae Hwang, John Jeka

**Affiliations:** 1 Spinal Cord Injury Research Laboratory, Crawford Research Institute, Shepherd Center, 2020 Peachtree Rd NW, Atlanta, GA 30309 USA; 2 Program in Biomechanics and Movement Science & Department of Physical Therapy, University of Delaware, 540 S. College Ave, Newark, DE 19713 USA; 3 Shriners Hospital for Children, 3551 North Broad Street, Philadelphia, PA 19140 USA; 4 University of Pennsylvania & The Children’s Hospital of Philadelphia, 3401 Civic Center Boulevard, Philadelphia, PA 19104 USA; 5 Department of Kinesiology, University of Maryland Eastern Shore, William P. Hytche Center Room 1124, Princess Anne, MD 21853 USA; 6 Department of Kinesiology, University of Delaware, 540 S. College Ave, Newark, DE 19713 USA

**Keywords:** Cerebral palsy, Balance, Postural control, Stochastic resonance stimulation, Somatosensation, Afferent stimulation

## Abstract

**Background:**

Stochastic Resonance (SR) Stimulation has been used to enhance balance in populations with sensory deficits by improving the detection and transmission of afferent information. Despite the potential promise of SR in improving postural control, its use in individuals with cerebral palsy (CP) is novel. The objective of this study was to investigate the immediate effects of electrical SR stimulation when applied in the ankle muscles and ligaments on postural stability in children with CP and their typically developing (TD) peers.

**Methods:**

Ten children with spastic diplegia (GMFCS level I- III) and ten age-matched TD children participated in this study. For each participant the SR sensory threshold was determined. Then, five different SR intensity levels (no stimulation, 25, 50, 75, and 90% of sensory threshold) were used to identify the optimal SR intensity for each subject. The optimal SR and no stimulation condition were tested while children stood on top of 2 force plates with their eyes open and closed. To assess balance, the center of pressure velocity (COPV) in anteroposterior (A/P) and medial-lateral (M/L) direction, 95% COP confidence ellipse area (COPA), and A/P and M/L root mean square (RMS) measures were computed and compared.

**Results:**

For the CP group, SR significantly decreased COPV in A/P direction, and COPA measures compared to the no stimulation condition for the eyes open condition. In the eyes closed condition, SR significantly decreased COPV only in M/L direction. Children with CP demonstrated greater reduction in all the COP measures but the RMS in M/L direction during the eyes open condition compared to their TD peers. The only significant difference between groups in the eyes closed condition was in the COPV in M/L direction.

**Conclusions:**

SR electrical stimulation may be an effective stimulation approach for decreasing postural sway and has the potential to be used as a therapeutic tool to improve balance. Applying subject-specific SR stimulation intensities is recommended to maximize balance improvements. Overall, balance rehabilitation interventions in CP might be more effective if sensory facilitation methods, like SR, are utilized by the clinicians.

**Trial registration:**

ClinicalTrials.gov identifier NCT02456376; 28 May 2015 (Retrospectively registered); https://clinicaltrials.gov/ct2/show/NCT02456376.

## Background

Control of human upright posture during standing and walking is critical for performing functional activities and requires the integration of sensory inputs from visual, vestibular, and somatosensory systems [1]. All these modalities are regulated dynamically and modified based on the individual, the performed task and the environmental conditions in a process also known as sensory reweighting [1]. For instance, the somatosensory system is predominant for maintaining balance on a static surface when vision is unavailable, whereas in a compliant surface the central nervous system (CNS) depends upon vestibular cues to regulate upright stance. Sensory impairments can influence postural control by either affecting sensory feedback during the execution of a motor response in a continuous changing environment or by experiencing difficulties in developing and pre-selecting the desired motor plan based on previous experience [1–3]. Therefore, the observed sensory dysfunction in approximately 90% of children with CP [4] may partially contribute to poor feedback and feedforward motor control [2], resulting in functional constraints associated with this pathology.

The development of movement and posture in CP is primarily affected by a static lesion that occurs in the developing fetal or infant brain [5]. Although the brain injury is not progressive, it results in motor and functional impairments that are progressive through a lifetime and related with reduced ambulatory ability [6] and poor balance performance [7–9]. In particular, postural instability has the most significant contribution to the model of primary impairments (i.e., related to the brain injury) compared to the muscle tone and motor coordination deficits in this population [10]. In contrast to normal postural responses, CP postural control characteristics include: descending pattern of muscle recruitment (proximal to distal strategy) [11], reverse order of muscle activation (antagonist followed by agonist activation) [11, 12], compensatory agonist/ antagonist co-activation [1], and inability to quickly modulate postural responses [1] and adapt to perturbations. Sensory deficits are also prevalent in individuals with CP [13–18], and can largely affect postural control and consequently balance. Furthermore, balance deficits in this population are associated with inability to successfully perform functional activities [1], increased risk for falls and higher levels of caregiver dependence [7], and can potentially lead to decreased chances for environmental exploration and social participation.

Postural control deficits in CP have been attributed in biomechanical changes in postural alignment and also in CNS and sensory processing impairments [19]. Disrupted thalamocortical networks [20, 21] and impaired somatosensory cortical activation [22–24] may affect motor behavior. This is consistent with our clinical findings on the relationship between plantar cutaneous and ankle proprioceptive impairments and motor deficits in CP [25]. Specifically, we provided evidence that aberrant plantar two-point discrimination, vibration sensation on the first metatarsal head, and ankle joint position sense were related with poor performance in the majority of the underlying systems that contribute to postural control as measured by BESTest and postural sway measures [25]. Damiano et al. (2013) showed significantly moderate to strong relationships between hip proprioceptive deficits in the transverse plane and balance parameters as measured during quiet bipedal stance with eyes open and eyes closed [26]. Altogether, these findings revealed the important link between lower extremities (LE) somatosensation with balance and motor ability, as somatosensory impairments affect both motor and balance control in individuals with CP. Therefore, the assessment and facilitation of LE somatosensation should be included in rehabilitation programs in this population.

Over the last decade, there is an increasing number of interventions targeting postural control and balance in children with CP [27]. A systematic review on postural control interventions identified only five training protocols that are potentially effective, based on a moderate level of evidence, and all of them are mainly motor-centric with the exception of hippotherapy which involves the provision of both sensory and motor cues through the horse’s movement [27]. Yet, this treatment is expensive and of limited availability [28]. The need for further sensory-oriented rehabilitation approaches has been previously highlighted in the literature [26, 29, 30], especially in light of evidence of plasticity in the white matter pathways following a combined therapy [31] and the potential of beneficial structural changes in the primary somatosensory cortex following somatosensory therapy in individuals with CP [32].

A promising sensory-centric therapeutic approach involves the modulation of somatosensory information by using a sub-sensory stochastic resonance (SR) stimulation to enhance balance control of upright stance. The phenomenon of SR, where random noise improves a nonlinear system’s sensitivity to differentiate a weak signal, has been observed in various biological systems [33, 34]. Specifically, in sensory systems, the presence of a low level of noise forces an undetectable weak signal (i.e., subthreshold sensory input) to cross the receptor’s sensory threshold and be detected; improving the system’s sensitivity [34]. Prior research has demonstrated improved behavioral performance in the presence of noise not only for subthreshold but also for suprathreshold sensory signals (i.e., suptrathreshold SR) [35]. Moreover, the effects of SR are present in the neuronal pathways of both the peripheral and CNS [36–40]. For instance, Iliopoulos et al. (2014) applied an unperceivable tactile stimulus and SR electrical noise into two distinct peripheral receptors and concluded that those two signals potentially converged in the CNS resulting in increased detectability of the weak tactile input [37]. Further, a number of studies showed that it is possible to apply noise within one sensory modality to enhance signal detection in another sensory modality, thus, suggesting that SR can play an important role in multisensory integration [38–40], an important component of postural control.

Mechanical or electrical SR stimulation has been used to enhance balance in: healthy adults [41–45], older people [41, 46], individuals with functional ankle instability [47, 48] and knee osteoarthritis [49], and patients with diabetic neuropathy and stroke [50] by improving the sensory signal’s strength in the somatosensory [41, 47–50] and vestibular [45, 51] systems. Recent evidence indicated that therapeutic interventions using electrical SR stimulation has ameliorated proprioceptive deficits and balance disturbances earlier and to a greater extent than traditional rehabilitation in individuals with ankle instability [52]. Conversely, a study by Kyvelidou and colleagues (2017) concluded that mechanical SR did not improve the development of sitting behavior when combined with a perceptual-motor intervention in children with CP between the ages of 2 to 6 years [53]. In this study, however, the intensity of the mechanical SR was determined upon the facial expressions of each participant (i.e., a therapist adjusted the amplitude of the vibratory tactors until the SR stimulus was not noticeable on the child’s facial expressions) potentially resulting in using inappropriate levels of SR noise that were not beneficial in advancing sitting postural control [53]. Furthermore, studies have suggested that an individualized level of SR stimulation can further improve balance performance [45, 51, 54, 55] than using the same predetermined SR intensity level for all participants.

The primary aim of this study was to investigate the immediate effects of SR electrical stimulation on balance performance in children and adolescents with CP. To ensure appropriate levels of SR stimulation during the balance task, we included in our experimental design a procedure to identify each participant’s SR sensory threshold followed by an optimization protocol to define the subject-specific optimal SR intensity [54]. We hypothesized that the application of SR would enhance balance control during quiet stance compared to a sham condition in individuals with CP. A secondary hypothesis proposed that children with CP would demonstrate greater improvements in balance performance compared to their typically developing (TD) peers when somatosensory SR stimulation is applied.

## Methods

### Participants

Ten individuals with CP and 10 age-matched TD peers between the ages of 8–18 years participated in this study. Children with CP were able to stand independently for at least 2 min (GMFCS I- III) and had a diagnosis of spastic diplegia. The inclusion and exclusion criteria are presented in Table 1. The protocol of this study was approved by the Western Institution Review Board (IRB) and the IRB of Temple University (for Shriners Hospital for Children, Philadelphia) and the University of Delaware. All the participants and their legal guardians signed the approved assent and consent documents, respectively.

**Table 1:** Inclusion and Exclusion criteria for participation in the study. Asterisk indicates the eligibility criteria met only by children with CP

Inclusion	Exclusion
● Age 8–18 years ● The diagnosis of spastic diplegic CP* ● Levels I-III GMFCS classification* ● Ability to stand independently (i.e., without using any assistive device) ● Visual, perceptual, and cognitive/ communication skills to follow multiple step commands ● Seizure-free or well controlled seizures ● Ability to communicate pain or discomfort during testing procedures ● Willingness to participate in testing ● Ability to obtain Parental/guardian consent and child assent/consent	● Diagnosis of athetoid, ataxic or quadriplegic CP* ● Significant scoliosis with primary curve > 40° ● Lower extremity surgery or fractures in the year prior testing ● Joint instability or dislocation in the lower extremities ● A history of selective dorsal root rhizotomy* ● Botulinum toxin injections in the lower extremities within the past 6 months* ● Marked visual, hearing, vestibular deficits ● Implanted medical device that may be contraindicated with application of SR stimulation ● Severe spasticity of any lower extremity muscle (e.g., a score of 4 on the Modified Ashworth Scale)* ● Pregnancy

### Experimental procedures

#### SR stimulation

**Fig. 1:**
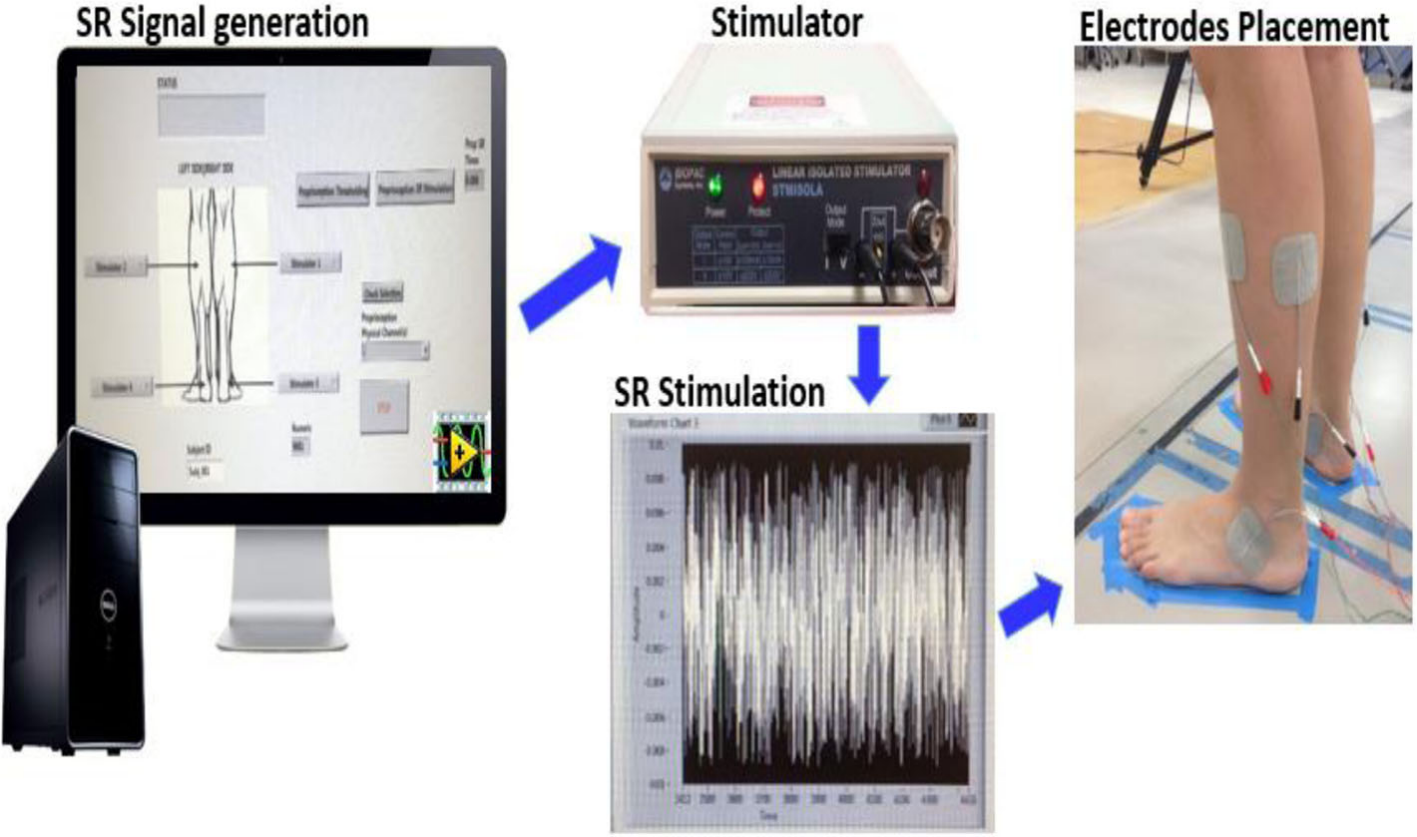
Schematic illustration of the SR Stimulation System. Our system consisted of a computer and 4 stimulators. The SR signal was generated by a custom LabView control program to trigger the stimulators that subsequently delivered electrical SR stimulation in the muscles and ligaments of the ankle joints

Our SR Stimulation System included four linear isolated stimulators (STMISOLA, Biopac Systems, Inc., Goleta, California, USA). SR stimulation consisted of white noise signal with a zero mean Gaussian (i.e., bell-shaped) distribution and bandwidth 0- 1000 Hz. This frequency range has been previously used to increase the sensitivity of proprioceptive receptors [49, 56] and adheres to the Moss et al. (2004) recommendation that the upper cut-off frequency of the noise signal should be significantly larger than the frequencies contained in the subthreshold sensory input [34]. White noise by definition has a flat power spectral density across the frequency range of interest. The SR signal was generated by a custom LabView program to trigger each Biopac stimulator via a 16 bit PCI 6733 National Instruments multifunction data acquisition card (Fig. 1). Self-adhesive stimulating electrodes, 5 × 5 cm, were placed over the lateral soleus, peroneus longus, and tibialis anterior muscles and anterior talofibular and deltoid ankle ligaments of each leg [48] after the skin was cleaned and dried. The aforementioned muscles and ligaments contribute to the ankle’s joint stability in both sagittal and frontal planes of motion. Flexible non-adhesive wrap (CoFlex, Andover Healthcare Inc., Salisbury, CA) was used to tightly secure the electrodes. The maximum current output, controlled by our LabView program, was limited to 5 mA.

#### SR sensory threshold

Initially, each individual’s sensory threshold (i.e., the level of stimulation required for an individual to just detect a tingling sensation on the stimulus sites) was identified [42, 54]. During the thresholding procedure, each subject was required to stand on both feet with their eyes closed, and the SR electrical stimulus amplitude was increased in 0.001 mA increments, initialized at zero, until the subject reported feeling the stimulation (SR sensory threshold). This threshold was verified if the subject could no longer perceive the stimulus when the intensity was decremented. This procedure was repeated four times and the lowest value indicated the subject’s sensory threshold and was recorded for subsequent reference.

#### SR experimental protocol

To investigate the effects of electrical SR stimulation in balance, four different stimulation intensities: 25, 50, 75, and 90% of the subject-specific sensory threshold and a sham, no stimulation, condition were used [54]. In line with previous studies that used electrical SR to improve signal detection [37, 49, 50, 56, 57], a range of 25 to 90% of the sensory threshold was chosen. All the tested intensities were below the sensory threshold as Severini and Delahunt (2018) showed that subthreshold SR is more effective in reducing postural sway compared to suprathrehold SR intensities [58]. Following the thresholding process, participants stood barefoot on 2 AMTI force plates (Advanced Mechanical Technology Inc.) in a standardized way [59]. For each trial, they were instructed to maintain a still and upright posture for 25 s while having their eyes open (EO) or closed (EC). During the eyes open condition, they were advised to keep their gaze straight ahead at the eye level, whereas during the eyes closed condition a sleeping mask was used to cover the eyes. For each stimulation intensity and visual feedback condition, two trials were performed. The no stimulation conditions were tested first and then the stimulation trials were performed in random order. Two additional no stimulation condition trials were performed at the end of the testing procedure to examine for learning or fatigue effects. The resting interval between trials depended on each participant’s comfort and fatigue level. Finally, an overhead harness system was used to prevent falls during each trial.

All the force plate data were collected using Vicon Nexus software (v1.8.5) at 100 Hz sampling rate. Also, to avoid any transient effects due to the addition of SR stimulation [60], only the last 20 s of each trial were filtered with a fourth-order, zero phase response, low-pass Butterworth filter with a cutoff frequency of 5 Hz [54, 61] and used for further analysis. To identify the optimal SR intensity, the differences between the resultant center of pressure velocity (COPVr) of the 4 SR stimulation conditions over the no stimulation condition were calculated. This measure has been previously used to determine the immediate effects of SR stimulation on balance [47, 54]. COPVr quantifies the magnitude of the COP displacement over time and it is a more reliable measure of postural steadiness compared to position and acceleration postural sway measures [62–65]. The intensity that produced the greatest reduction in COPVr (i.e., suggesting improved balance performance) was defined as the optimal SR intensity for each individual and was subsequently used for the analysis. This optimization procedure has been previously described by Ross and colleagues (2013) for efficient and timely determination of the SR intensity levels [54]. Then, the COPV in A/P and M/L directions, COPA, and RMS A/P ad M/L distance of COP displacement for the optimal SR stimulation and no stimulation conditions were computed. These COP-based measures have been previously used to determine the effect of SR stimulation during upright stance in individuals with functional ankle instability [54].

### Statistical analysis

All data were analyzed using SPSS (version 23; SPSS Inc., Chicago. IL, USA) with the level of significance set at *p* < 0.05. Initially, the data were examined for normality using Shapiro-Wilk test and Q-plots. All the data were normally distributed except the COPA measures that were transformed with a square root transformation before proceeding with the analysis. Means and standard errors were calculated for the demographic data and all the COP variables of interest.

Independent samples t-tests were performed to examine if there were significant differences between groups in age, height, weight, and BMI. A Fisher’s exact test determined if there were sex differences. To rule out learning or fatigue effects, paired t-tests were computed on the COPVr of the initial and last no stimulation trials for EO and EC conditions. Separate one-way repeated measures of ANOVA were used to identify which stimulation intensity (i.e., 25, 50, 75%, or 90% of SR sensory threshold) produced the greatest reduction in the COPVr for eyes open and closed conditions.

#### Effects of SR stimulation on balance in the CP group

To investigate the effects of SR stimulation in the CP group, all the COP measures were examined separately by a two-way repeated measures ANOVA with 2 within factors (intensity: optimal SR stimulation, no stimulation condition; visual feedback: EO, EC). Based on our a priori hypothesis, Bonferroni-adjusted paired t-tests for planned comparisons were performed between the optimal SR stimulation and no stimulation conditions when a significant main effect for intensity condition was found.

#### Comparison of SR stimulation effects on balance in the CP versus the TD group

Independent samples t- tests were used to assess the differences between the two groups on the SR sensory threshold and the SR optimal intensity. To investigate if children with CP demonstrated greater improvements in their balance performance when SR stimulation was applied compared to their TD peers, the differences between the COP measures of the optimal stimulation intensity over the no stimulation condition were computed, square root transformed, and subsequently used for the analysis. Separate 2 × 2 mixed model repeated measures ANOVA with visual feedback (EO, EC) as the within-subjects factor and group (CP, TD) as the between-subjects factor were conducted for the COP measures. Bonferroni corrected planned comparisons (unpaired t-tests) were performed between the CP and TD groups for each visual feedback condition when a significant group effect was found.

## Results

All children completed the experimental process, however, due to technical problems during the collection of the kinetic data, only the data of 18 participants (9 CP and 9 TD children) were analyzed. No significant differences were found for age, sex, height, weight, and BMI between the CP and the TD groups (Table 2).

**Table 2:** Demographic characteristics of children with cerebral palsy and their typical developing peers

	CP group (*n* = 9)	TD group (n = 9)
Age (years, months)	15 y 5 mo (1y 0.9 mo)	15 y 6 mo (0.8 y 1.3 mo)
Sex (male/ female)	8/ 1	5/ 4
GMFCS (level)	I: 3; II: 3; III: 3	–
Height (cm)	164 (3.9)	164.9 (5.4)
Weight (kg)	66 (9.9)	60.4 (8.4)
BMI (kg/m^2^)	23.5 (2.6)	21.5 (1.8)

Means and standard errors (in parentheses) are presented in the table

Paired sample t-tests on the initial and the last no stimulation conditions for the COPVr measure showed no significant differences and thus, ruled out any fatigue or carry over effects of SR stimulation (EO: t(17) = 1.4, *p* = 0.18; EC: t(17) = 0.76, *p* = 0.46). A repeated measures ANOVA determined that one out of the 4 SR tested intensities (i.e., optimal SR intensity) significantly decreased the COPVr (F(3, 24) = 6.97, *p* < 0.002, partial η^2^ = 0.47). This finding supported the importance of identifying the optimal SR intensity for improving balance.

### Effects of SR stimulation on balance in the CP group

Separate 2 (intensity) X 2 (visual feedback) repeated measures ANOVAs were conducted to examine the effectiveness of SR on balance in children with CP (Table 3). A main effect was obtained for intensity, with participants demonstrating decreased COPV in A/P and M/L directions, and COPA measures when optimal SR stimulation was applied (*p* < 0.05). Additionally, a main effect was found for visual feedback condition only for the COPV in M/L direction indicating that when participants with CP had their eyes closed they exhibited higher COP velocity in the frontal plane (F(1,8) = 5.61, *p* = 0.04, partial η^2^ = 0.41). Finally, we did not find any main effects for the RMS measures or a significant intensity X visual feedback interaction for all the tested COP measures.

**Table 3:** Main effects of intensity (no stimulation vs. SR optimal stimulation) and visual feedback (eyes open vs. eyes closed) for the COP measures in children with CP

Repeated Measures Two-way ANOVA						
	Main Effect: Intensity			Main Effect: Visual Feedback		
	F value	*P* value	Partial η^2^	F value	*P* value	Partial η^2^
COPV A/P (cm/s)	F(1,8) = 5.56	p = 0.04	0.41	F(1,8) = 0.01	*p* = 0.90	0.00
COPV M/L (cm/s)	F(1,8) = 7.54	*p* = 0.02	0.81	F(1,8) = 5.61	p = 0.04	0.41
COPA (cm^2^)	F(1,8) = 6.96	*p* = 0.03	0.45	F(1,8) = 0.00	*p* = 0.99	0.00
RMS A/P (cm)	F(1,8) = 1.95	*p* = 0.20	0.19	F(1,8) = 0.19	*p* = 0.67	0.02
RMS M/L (cm)	F(1,8) = 5.35	p = 0.07	0.35	F(1,8) = 0.58	*p* = 0.47	0.07

**Fig. 2:**
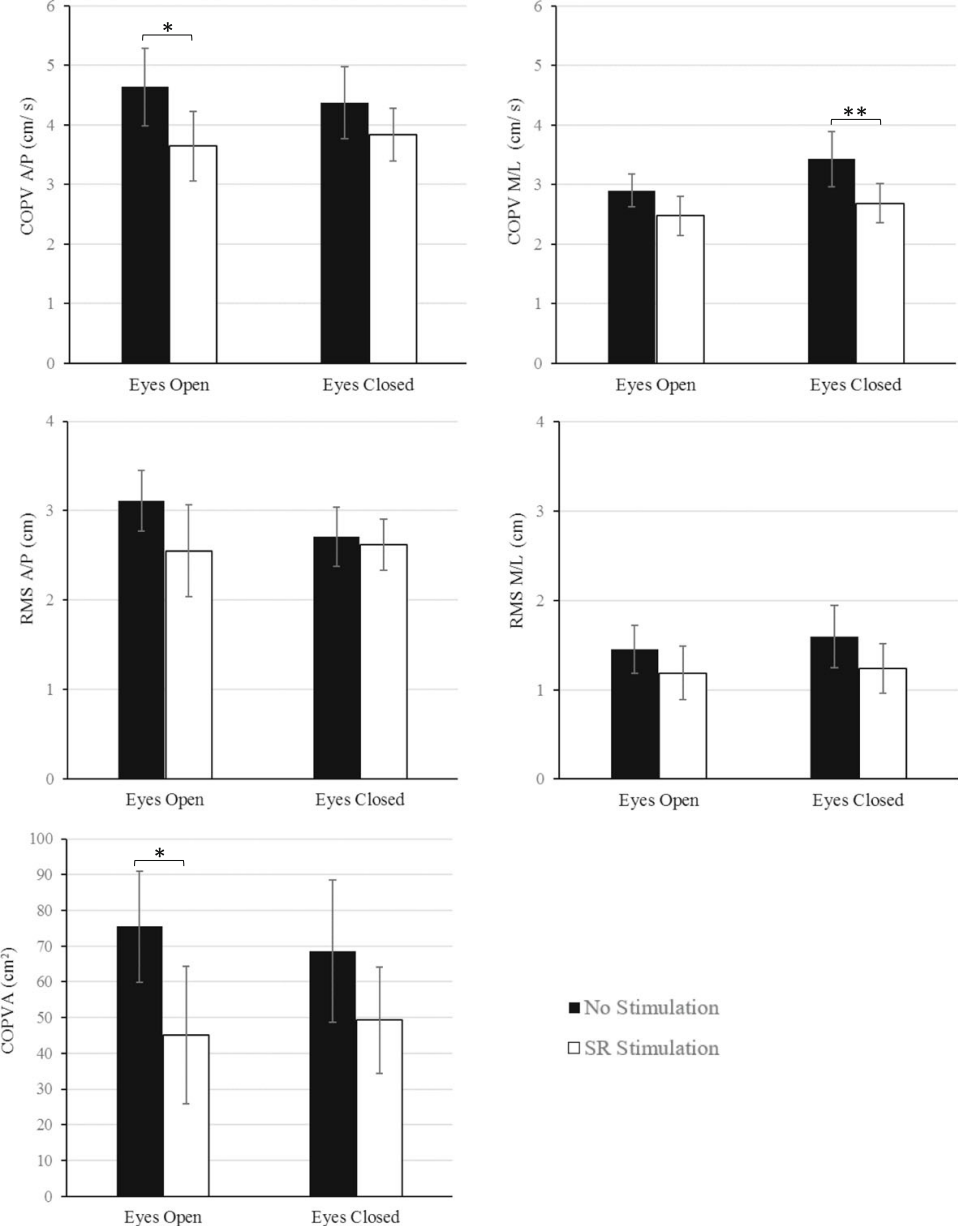
Center of Pressure measures during upright quiet stance in children with cerebral palsy with their eyes open and closed. White bars represent the control-no stimulation-condition and the black bars the optimal SR stimulation condition. Error bars represent standard errors, * *p* < 0.05, ** *p* < 0.01

Figure 2 shows means and standards errors for all the COP measures for both eyes open and eyes closed conditions. Specifically, children with CP improved their balance with the addition of the optimal SR stimulation compared to the no stimulation condition for all measures. These improvements were significant only for the COPV in A/P direction, and COPA measures for the eyes open condition and for the COPV in M/L direction and measures for the eyes closed condition.

### Comparison of SR stimulation effects on balance in the CP versus TD group

**Fig. 3:**
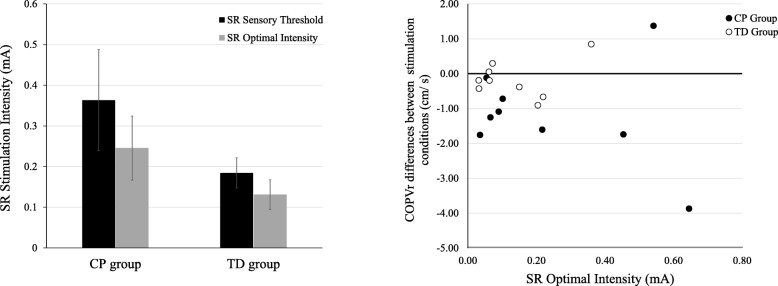
(**a**) SR sensory threshold and SR optimal intensity mean values for CP and TD groups. Black bars represent the SR sensory threshold and gray bars the SR optimal stimulation intensity. Error bars represent standard errors. Independent samples t-tests did not reveal significant differences between the two groups. (**b**) Scatter plot of the SR optimal intensity level and the difference between the COPVr of the optimal SR intensity over the no stimulation condition. Each black data point reflects a child with CP and each white data point reflect a child with TD. The black line is crossing y-axis at 0 and represents no change in the COPVr data following the application of SR stimulation. Data points falling above the black line suggest diminished postural sway, whereas those below the line suggest improved postural sway

Mean SR sensory threshold and SR optimal intensity levels were not significantly different between groups (Fig. 3A; SR sensory threshold: t(16) = 1.38, *p* = 0.19; SR optimal intensity: t(16) = 1.31, *p* = 0.21). Figure 3B presents a plot of the SR optimal intensity level and the difference between the COPVr of the optimal SR intensity over the no stimulation condition for all participants. Specifically, the addition of SR stimulation diminished balance performance in 1 child with CP and 3 children with TD whereas the remaining participants responded positively to the application of SR stimulation since their balance improved.

To investigate if children with CP demonstrated greater balance improvements than their TD peers due to the application of SR, separate 2 (intensity) X 2 (group) mixed model repeated measures ANOVAs, with intensity as a within factor were conducted for all the COP variables of interest. A significant main effect was found for group in COPV in M/L direction (F(1,16) = 7.37, *p* = 0.01, partial η^2^ = 0.32), and COPA (F(1,16) = 8.52, *p* = 0.01, partial η^2^ = 0.35). Additionally, the group main effect for COPV (F(1,16) = 3.78, *p* = 0.07, partial η^2^ = 0.19) and RMS (F(1,16) = 3.9, *p* = 0.06, partial η^2^ = 0.20) in A/P direction approached significance. These results indicated that the CP group benefited more from the application of SR during upright stance than the TD group. Furthermore, the planned comparisons suggested that children with CP significantly improved balance compared to the TD group when visual information was provided (Table 4). For the eyes closed condition, the CP group showed significantly greater balance performance with the SR noise compared to controls only for the COPV in M/L direction (Table 4).

**Table 4:** Mean ± SE for the differences between the COP measures of the optimal SR stimulation intensity over the no stimulation condition for children with CP and their TD peers

	Eyes Open		Eyes Closed	
	CP Group	TD Group	CP Group	TD Group
COPV A/P (cm/s)	−0.99 ± 0.40^*^	0.05 ± 0.29	−0.54 ± 0.38	0.01 ± 0.28
COPV M/L (cm/s)	−0.42 ± 0.32^**^	0.20 ± 0.20	− 0.75 ± 0.24^*^	0.14 ± 0.33
COPA (cm^2^)	−30.25 ± 18.84^**^	0.12 ± 2.9	−19.34 ± 15.13	0.54 ± 5.02
RMA A/P (cm)	−0.56 ± 0.30^**^	0.08 ± 0.17	−0.09 ± 0.29	0.18 ± 0.20
RMS M/L (cm)	−0.26 ± 0.27	0.13 ± 0.09	−0.36 ± 0.20	− 0.01 ± 0.16

The negative sign indicates that the addition of SR resulted in decreased COP measures suggesting balance improvements. Increased COP measures demonstrate diminished balance performance

Asterisks denote significant differences between groups for each visual feedback condition (* *p* < .05; ** *p* < .01)

**Fig. 4:**
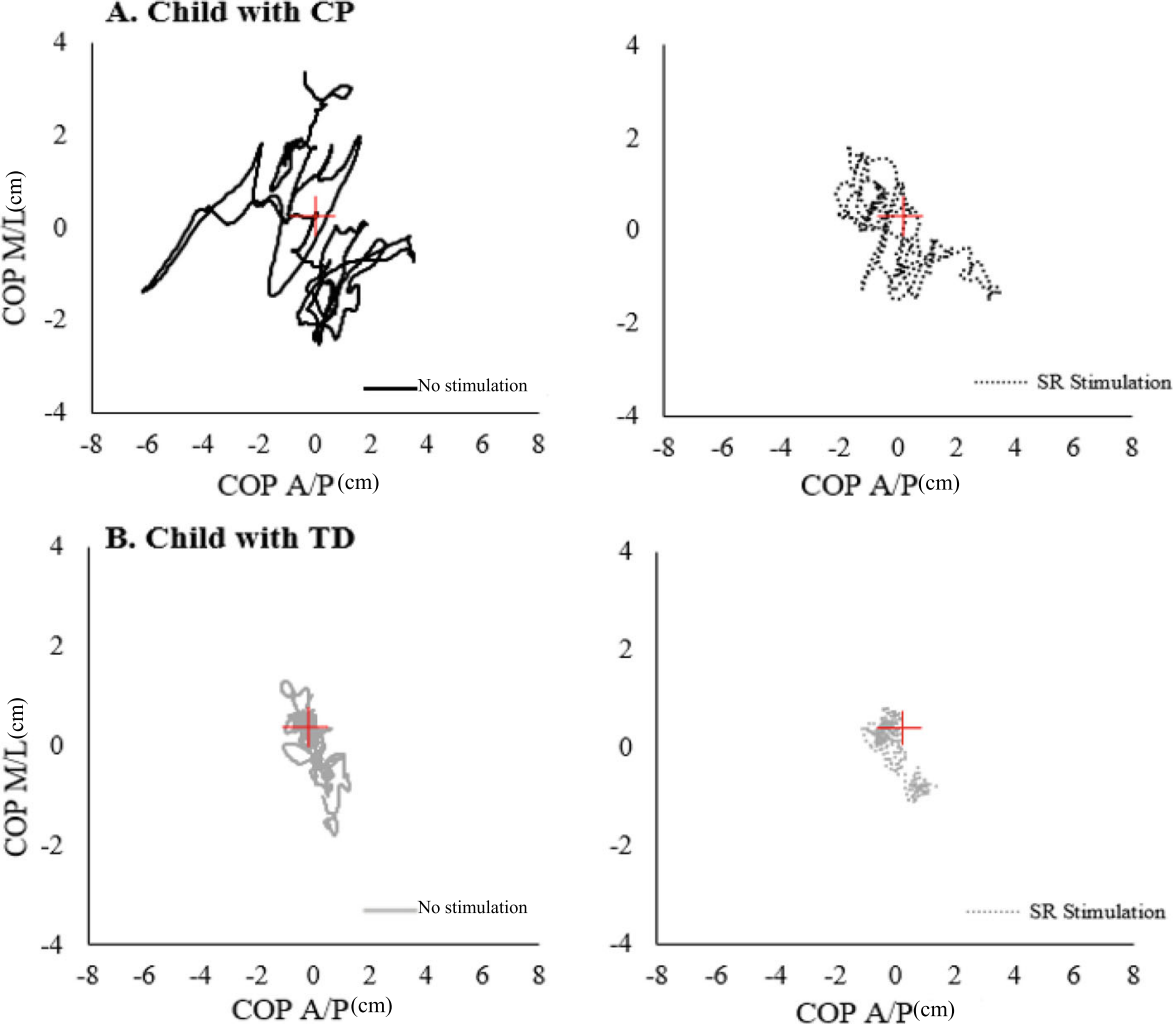
Representative data from a child with CP (**a**) and a TD individual (**b**), showing center of pressure stabilograms during quiet stance with their eyes open. Two experimental condition are shown for: control-no stimulation-condition (solid line), and SR Optimal Stimulation condition (dotted line). In the optimal SR stimulation condition the 95% confidence ellipse area of the COP sway was decreased by 16.53mm^2^ for the child with CP and by 0.91mm^2^ for the TD child compared to the no stimulation condition

In Fig. 4, representative stabilograms for a child with CP and a child with TD showed COP sway traces during quiet stance for both the SR stimulation and no stimulation conditions. The graphs showed that adding an optimal SR noise decreased the area of the COP sway for both participants, thus indicating improved postural stability. The decrease, however, was larger for the child with CP.

## Discussion

In this study, we investigated the immediate effects of SR stimulation during quiet stance in individuals with CP and their TD peers. Specifically, we showed that applying a subsensory SR noise in the muscles and ligaments of the ankle joints during quiet stance resulted in decreased COP sway compared to the control-no stimulation-condition in the CP group. Additionally, we demonstrated that the improvements in balance performance (i.e., reductions in the COP measures) due to the application of SR were significantly greater in the CP group than the TD group. Overall, the detected balance improvements were potentially due to the upregulation of the afferent somatosensory inputs, as the SR stimulation increased their detectability by the CNS and enhanced sensory integration. These findings suggested that SR stimulation is a promising tool that, upon further development, can be used as part of future therapeutic interventions for the treatment of balance deficits in children with CP.

Previous studies showed that applying SR noise in the lower extremities can decrease postural sway and improve balance in populations with somatosensory deficits [41, 48–50, 54, 66]. Likewise, we demonstrated that children with CP, who exhibit foot and ankle somatosensory deficits, can benefit from the application of electrical SR noise in the lower extremities during standing. One potential neurophysiological mechanism that describes electrical SR is that the subthreshold electrical noise signals cause small changes in receptor transmembrane potentials, which, in turn, make the sensory neuron more likely to fire an action potential in the presence of a weak stimulus [41, 50]. Studies investigating the effects of electrical SR on balance used a broad frequency range (0-2000 Hz) of the subthreshold noise signal to improve the sensitivity of proprioceptive receptors [42, 49, 58]. We speculated that high frequency electrical SR noise enhanced the excitability of both muscle spindles and joint receptors of the targeted muscle groups and ligaments. This resulted in lower proprioceptive receptors thresholds and, thus, increased detectability of afferent signals by CNS. Integration of these inputs by CNS allowed for improved postural reflexes and subsequently balance function in CP, as a stable standing position mainly depended on ankle joint proprioception [67].

Our results demonstrated that the application of SR resulted in significant balance improvements in individuals with CP primarily in the eyes open condition. Due to their inherent somatosensory impairments, it is possible that children with CP relied more on their visual input to maintain a stable upright stance. This is not surprising since previous research showed visual dependency as a compensatory strategy for proprioceptive deficits in CP [17]. Conversely, children with CP showed similar postural sway increments when vision was occluded as the control group, indicating that they did not have to depend more on visual feedback to maintain upright stance [68]. In our study, we speculated that the upregulated somatosensory information, due to the addition of the SR noise, and along with the visual information, provided enhanced sensory inputs and processing that resulted in improved balance control compared to the eyes closed condition. Additionally, based on the crossmodal SR phenomenon [38, 39], the application of noise in the somatosensory system potentially increased visual input, thus resulting in improved multisensory integration. Research suggested that the stronger sensory inputs and enhanced sensorimotor integration that were elicited by noise can contribute to improved motor performance and this improvement is consistent with an increase in cortical motor spectral power and corticomuscular coherence [69]. Especially for individuals with CP, whose neurological insult took place prior to having the ability to learn flexible and stable movement, improving not only the afferent signal but also sensory processing and corticospinal drive may have significant clinical implication in balance rehabilitation.

An important characteristic of SR is the inverted U-shaped relationship between signal’s detectability and the noise’s intensity [33, 36, 37, 55]. According to this relationship, there is an optimal level of noise that results in maximal detectability of a weak signal. Further, the optimal level of noise can be below or above the sensory SR threshold. Trenado and colleagues (2014) demonstrated that low frequency subthreshold and high frequency suprathreshold mechanical SR noise can improve the performance of a sensorimotor task [70]. On the other hand, subthreshold over suprathreshold electrical SR noise enhanced tactile perception in healthy adults [37]. For balance improvements, previous studies have used SR noise levels below sensory threshold. In a comparative study, the researchers concluded that subthreshold levels of noise are superior in improving postural balance compared to suprathreshold levels of noise [58]. In our study, we applied subsensory electrical SR noise during quiet stance that resulted in decreased postural sway in individuals with CP. Hence, it is suggested that future research investigates if suprathrehold SR noise can influence balance performance in this population.

To determine the optimal subthreshold level of noise, an optimization procedure was followed. Specifically, from a broad range of SR intensities (25–90% of sensory threshold) we identified the optimal intensity level for decreased postural sway. This procedure is critical as higher or lower SR intensity levels can degrade performance. Similar SR optimization protocols have been previously used to enhance somatosensory [42, 54] and vestibular information [45, 51] to improve postural stability. On the contrary, using an unreliable procedure to define the SR threshold, as previously described in the introduction section, did not produce any increase in advancing sitting behavior in children with CP [53]. Altogether, determining the subject-specific optimal SR intensity is a crucial component of SR testing to maximize somatosensory signal’s detectability and to subsequently improve balance function. From a clinical standpoint, using a simple and time efficient procedure to identify the appropriate levels of SR stimulation can advance the therapeutic benefits of SR noise in balance training protocols in individuals with postural control deficits similar to our participants.

Another important consideration regarding the application of SR is that the externally applied noise also depends upon the levels of the internal noise [36]. Internal SR noise is present in every level of the nervous system, from the cellular excitability to the execution of a motor task [71], and its intensity varies not only across subjects but also within the same subject [36]. Aihara et al. (2010) suggested that when the internally generated noise is already at high levels, then the addition of external noise may diminish performance and vice versa [36]. In line with this notion, our findings showed that in 4 participants (one child with CP and 3 children with TD) external SR noise diminished postural sway potentially due to the fact that these individuals exhibited higher internal noise levels. When comparing the group means, children with CP improved their standing balance with the addition of SR whereas their TD peers did not. Moreover, the CP group had higher SR sensory thresholds and SR optimal intensity levels compared to the TD group, however, the difference between the groups was not significant. We speculated that individuals with CP had lower levels of internal noise and the application of the external SR noise facilitated the somatosensory signal detection resulting in improved balance. In addition, the internal noise levels differed within the CP group as for each individual a different optimal SR intensity was identified (see Fig. 3.B for reference). This increased variability can be attributed to the heterogeneity of the CP group as participants with CP in this study exhibited a broad range of functional ability- from being able to walk independently (GMFCS I) to using wheeled mobility in the community and for longer distances (GMFCS III)- indicating large variations in both motor and sensory function. To the contrary, TD group potentially exhibited higher internal noise levels and applying SR on their lower extremities during quiet stance either slightly improved or attenuated their postural stability. Future studies should further our understanding on how CP might influence the levels of internal noise in the nervous system and the interplay between internal and external noise to enhance sensory information processing and movement.

In this study, we assessed postural control by using linear COP measures during a simple standing paradigm to characterize postural stability in children with CP. Although these measures have been used before to test the effects of SR stimulation on standing balance in different populations with diminished somatosensory ability [41, 42, 44], they only provide information on the displacement of COP in A/P and M./L dimension. Since postural control is a complex motor skill that involves the integration of multiple sensory systems, future studies should examine how electrical SR stimulation can influence multisensory fusion during upright stance in order to provide insights into the dynamic characteristics of COP displacement in this population.

We acknowledge that our findings were interpreted in light of the assumption that a decrease in the computed COP variables due to the application of SR indicated balance improvements. In agreement, prior studies on postural balance in CP indicated that decreased COP sway is associated with increased stability [7, 9, 26, 68]. Specifically, and similar to our COP outcome measures in the no stimulation condition between the 2 groups, children with CP usually demonstrate increased postural sway during quiet stance compared to their TD peers. Some factors that can potentially contribute to the increased postural sway include, but are not limited to, poor postural alignment, abnormal muscle tone, sensory processing impairments, and diminished lower extremity somatosensory ability (i.e., larger COP oscillation are related with ankle joint rotations and, hence, greater activation of the proprioceptive receptors in an effort to collect more somatosensory information and compensate for the somatosensory deficits) [1]. Based on previous literature [1, 12] and empirical data from our lab, however, some individuals with CP may utilize co-activation of the agonist and antagonist muscle groups as a compensatory strategy to maintain their upright stance and exhibit a stiff posture. For example, Burtner et al. (1998) showed that children with spastic CP demonstrate significant coactivation of their lower extremities muscles during recovery of balance following backward platform perturbations to potentially stabilize the involved joints for improved postural control [12]. In this subgroup of individuals, decreased postural sway would suggest balance impairments, and inability to adapt in a constantly changing dynamic environment. For this reason, identifying the individuals with CP that share common postural control strategies can be useful in designing appropriate treatment plans to address balance deficits.

## Conclusion

Rehabilitation interventions in CP have thus far focused on improving motor performance but with limited consideration of somatosensory impairments, whose deficits can affect motor behavior. In addition, there is no universally-accepted framework for the identification of sensory processing impairments in children with developmental disorders, thus resulting in misdiagnosis and eventually in poor treatment [72]. Since somatosensory information is a key component of postural and motor control, more comprehensive clinical sensory assessments and more effective interventions should include sensory facilitation methods, like SR stimulation, as part of the everyday treatment procedure.

Our findings showed that SR stimulation can potentially be used as a therapeutic tool to improve balance performance by upregulating somatosensory information in children with CP. Clinicians and researchers who plan to utilize SR stimulation to modulate somatosensory input should apply subject-specific SR intensities to maximize balance improvements. Training protocols that combine afferent SR stimulation while performing daily activities may promote neuroplasticity [73] and, as a result enhance motor and sensory function compared to traditional motor-centric protocols.

## Abbreviations

A/P: Anteroposterior
CNS: Central Nervous System
COP: Center of Pressure
COPA: 95% COP Confidence Ellipse Area
COPV: Center of Pressure Velocity
COPVr: Resultant Center of Pressure Velocity
CP: Cerebral Palsy
EC: Eyes Closed
EO: Eyes Open
GMFCS: Gross Motor Function Classification Scale
LE: Lower Extremities
M/L: Medial-lateral
RMS: Root Mean Square
SR: Stochastic Resonance
TD: Typical Developing
